# Fluorescent-Tagged Antiscalants—The New Materials for Scale Inhibition Mechanism Studies, Antiscalant Traceability and Antiscaling Efficacy Optimization during CaCO_3_ and CaSO_4_·2H_2_O Scale Formation

**DOI:** 10.3390/ijms24043087

**Published:** 2023-02-04

**Authors:** Sergey Tkachenko, Maria Trukhina, Anastasia Ryabova, Maxim Oshchepkov, Semen Kamagurov, Konstantin Popov

**Affiliations:** 1 Department of Chemical and Pharmaceutical Technologies and Biomedical Pharmaceuticals, Mendeleev University of Chemical Technology of Russia, Miusskaya Sq. 9, 125047 Moscow, Russia; 2 JSC “Fine Chemicals R&D Centre”, Krasnobogatyrskaya, Str. 42, b 1, 107258 Moscow, Russia; 3 Prokhorov General Physics Institute of the Russian Academy of Sciences, Vavilov Str., 38, 119991 Moscow, Russia

**Keywords:** scale inhibition, fluorescent antiscalants, antiscalant visualization, gypsum, calcium carbonate, aminomethylenephosphonate

## Abstract

Equipment scaling leads to reduced production efficiency in a wide range of industrial applications worldwide. Various antiscaling agents are currently commonly used to mitigate this problem. However, irrespective of their long and successful application in water treatment technologies, little is known about the mechanisms of scale inhibition, particularly the localization of scale inhibitors on scale deposits. The lack of such knowledge is a limiting factor in the development of applications for antiscalants. Meanwhile, fluorescent fragments integrated into scale inhibitor molecules have provided a successful solution to the problem. The focus of this study is, therefore, on the synthesis and investigation of a novel fluorescent antiscalant: (2-(6-morpholino-1,3-dioxo-1H-benzo[de]isoquinolin-2(3H)yl)ethylazanediyl)bis(methylenephosphonic acid) (ADMP-F) which is an analog of the commercial antiscalant: aminotris(methylenephosphonic acid) (ATMP). ADMP-F has been found to effectively control the precipitation of CaCO_3_ and CaSO_4_ in solution and is a promising tracer for organophosphonate scale inhibitors. ADMP-F was compared with two other fluorescent antiscalants—polyacrylate (PAA-F1) and bisphosphonate (HEDP-F)—and was found to be highly effective: PAA-F1 > ADMP-F >> HEDP-F (CaCO_3_) and PAA-F1 > ADMP-F > HEDP-F (CaSO_4_·2H_2_O). The visualization of the antiscalants on the deposits provides unique information on their location and reveals differences in the “antiscalant-deposit” interactions for scale inhibitors of different natures. For these reasons, a number of important refinements to the mechanisms of scale inhibition are proposed.

## 1. Introduction

Scale is a solid layer deposited on the surfaces of industrial equipment through a process called scaling [1]. Scaling leads to increased costs and reduced production efficiency in reverse osmosis facilities, boilers and heat exchangers, evaporation plants, industrial water systems, geothermal power plants and oilfield applications [1,2,3,4]. In this regard, a variety of chemical [4] and nonchemical [5] techniques to mitigate scaling have been adopted. The simple operation and excellent scale resistance of antiscalants make them very popular [3,4,6,7,8]. The most efficient scale inhibitors include organophosphonates (aminotris(methylenephosphonic acid), ATMP; 1-hydoxyethylydene-1,1-bisphosphonic acid, HEDP; etc.) and polycarboxylates (polyacrylic acid, PAA; polyepoxysuccinic acid, PESA; polyaspartic acid, PASP; etc.) [6,7,8]. However, the questions of (i) the optimal selection of antiscalants for specific cases, (ii) the effective “on-line” control of their concentration in industrial plants, and (iii) the mechanisms of scale inhibition are still being discussed, notwithstanding their long, intensive and successful application in industrial water treatment technologies [2,4,8,9]. In the latter case, there is a general agreement that scale inhibitors can act in a bulk aqueous medium simultaneously by several possible routes [2,4,6,8,9]. They may: (i) increase scale solubility by masking cations such as Ca^2+^ and Ba^2+^ by the formation of soluble complexes; (ii) adsorb onto sparingly soluble salt nuclei (this reduces the rate at which the nucleus overcomes the critical size barrier (the “threshold effect”) and thus, reduces the rate of scale nucleation); (iii) impart a significant electrostatic charge to the scale nuclei, retarding their aggregation by electrostatic repulsion between sparingly soluble salt colloids; and (iv) adsorb onto growing crystals and block the crystal growth centers (this sometimes changes the crystal habit and slows down further crystal growth). However, the contribution of the specific mechanism in specific cases (evaporation, reverse osmosis, oilfield facilities, etc.) is usually unclear [9]. On the other hand, the conventional laboratory methods for testing the efficacy of antiscalants are imperfect and frequently lead to failure in industrial-scale applications due to the lack of sufficient knowledge of the corresponding inhibition mechanism [8].

Most of these problems could be successfully solved by the application of fluorescent-tagged antiscalants, which have gained increasing interest over the past decades [9,10,11,12,13,14,15,16,17,18,19,20,21,22,23,24,25,26,27,28]. Indeed, the precise monitoring of polycarboxylates is a challenge for water treatment technologies. Fluorescence detection has therefore attracted increasing attention as a powerful tool in chemical sensing due to its high sensitivity and simplicity [13,14,15,18,19,20,21,22,23,24,25,26,27,28,29]. However, the “online” control of phosphonate concentration in water recirculation systems is also an actual issue [11,30]. At the same time, fluorescent antiscalants provide a unique opportunity to visualize the location of the antiscalant during scale deposition and to gain an in-depth understanding of scale inhibition mechanisms. Remarkably, no reports on the visualization of fluorescently tagged scale inhibitor molecules have been registered up to 2019. Meanwhile, the first communication focused on fluorescent-tagged bisphosphonate HEDP-F (1-hydroxy-7-(6-methoxy-1,3-dioxo-1H-benzo[de]isoquinolin-2(3H)-yl)heptane-1,1-diyldi(phosphonic acid)) localization during gypsum deposition [15] already detected a paradoxical effect that conflicted the conventional theory of scale inhibition [4,9]. It was found that contrary to popular belief, HEDP-F does not interact at all with CaSO_4_·2H_2_O crystals at ambient temperature but retards gypsum scaling from supersaturated solution in a batch test [15]. Later the similar effect was reported independently for both static and dynamic conditions of gypsum crystallization in the presence of HEDP-F and fluorescent-tagged polymers [14,16,21]. This effect was explained in terms of “antiscalant—solid nanoimpurities” interactions [9]. At the same time, it was noticed that in a static test according to NACE recommendations (24 h heating of supersaturated gypsum solution at 70 °C [31]), HEDP-F was found both inside and on the surface of CaSO_4_·2H_2_O crystals [15]. Thus, the mechanism of gypsum scale inhibition at ambient and elevated temperatures appears to be different. This, in turn, raises questions about the validity of the NACE test for experimental conditions other than those specified by NACE.

**Figure 1:**
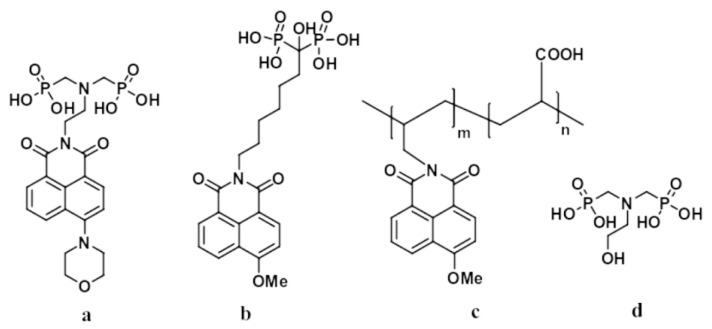
Chemical structures of ADMP-F (**a**), HEDP-F (**b**), PAA-F1 (**c**) and HEMPA (**d**).

Therefore, the development and testing of new fluorescent markers for specific water treatment applications are of undoubted interest. An evident gap here is the lack of fluorescent-tagged aminophosphonic antiscalants. Therefore, this study is focused on the synthesis and testing of a novel antiscalant: (2-(6-morpholino-1,3-dioxo-1H-benzo[de]isoquinolin-2(3H)-yl)ethylazanediyl)bis(methylenephosphonic acid) (ADMP-F) (Figure 1a). ADMP-F is a conjugate of aminobis(methylenephosphonic acid) (the closest structural analog to the highly effective antiscalant ATMP [8]) and a naphthalimide moiety, known as a highly effective fluorescent probe [32]. In addition, a recent communication by Rabizadeh [33] showed the high antiscaling efficacy of hydroxyethylamino-di(methylene phosphonic acid) (HEMPA) (Figure 1d), the closest structural analog of ADMP-F. In this study, ADMP-F is tested along with two other fluorescent antiscalants previously synthesized by our group: HEDP-F [14] and naphthalimide-tagged polyacrylate PAA-F1 [24] (Figure 1b,c) as well as the commercial antiscalant Aminat-K with ATMP as an active component. To the best of our knowledge, this is the first communication on the properties and visualization of ATMP and HEMPA fluorescent analogs.

A set of fluorescent scale inhibitors of different types (aminophosphonate (ADMP-F), alkylbisphosphonate (HEDP-F), polyacrylate (PAA-F1)) was tested in this work under laboratory conditions, reproducing the formation of calcium carbonate scale and gypsum deposits in evaporation plants, and compared, where possible, with the results of NACE tests previously obtained for HEDP-F and PAA-F1 [15,24]. Most importantly, unlike the NACE test, where supersaturation of sparingly soluble salts was achieved “all at once” immediately after the cation and anion brine were mixed, our experiments provided the more common situation, where supersaturation occurs gradually from initially unsaturated solutions. 

The key objectives of the present study are: (i) to evaluate the efficacy of a novel fluorescent antiscalant against calcium carbonate and gypsum deposition in comparison with two other fluorescent inhibitors of different natures; (ii) to gain insight into the inhibition mechanisms through visualization of scale inhibitors; and (iii) to compare the efficacy of antiscalants, estimated within the framework of different tests, based on different ways of achieving supersaturation.

## 2. Results

### 2.1. Synthesis of ADMP-F

**Scheme 1:**
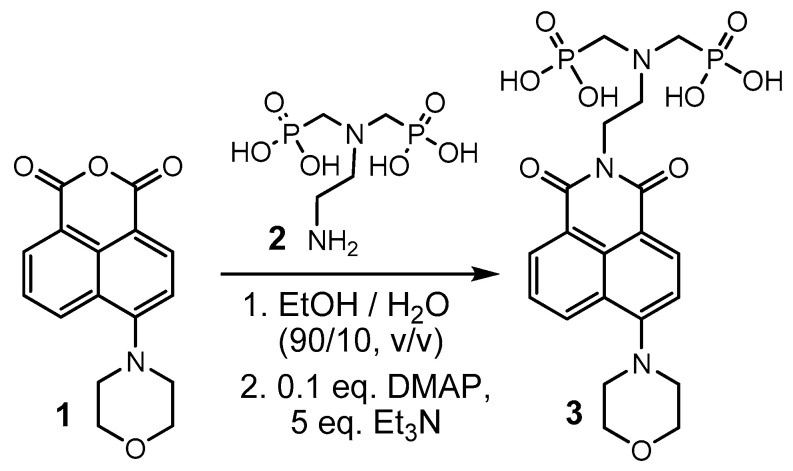
General scheme of ADMP-F synthesis. Compound 1, (6-morpholinobenzo[de]isochromene-1,3-dione); Compound 2, [[(2-aminoethyl)imino]bis(methylene)]bis(phosphonic acid); Compound 3, (2-(6-morpholino-1,3-dioxo-1H-benzo[de]isoquinolin-2(3H)-yl)ethylazanediyl)bis(methylenephosphonic acid) (ADMP-F).

The synthesis of (2-(6-morpholino-1,3-dioxo-1H-benzo[de]isoquinolin-2(3H)-yl)ethylazanediyl)bis(methylenephosphonic acid) (ADMP-F, Compound 3) was performed according to Scheme 1, the starting materials used were (6-morpholinobenzo[de]isochromene-1,3-dione) (Compound 1) and [[(2-aminoethyl)imino]bis(methylene)]bis(phosphonic acid) (Compound 2).

**Figure 2:**
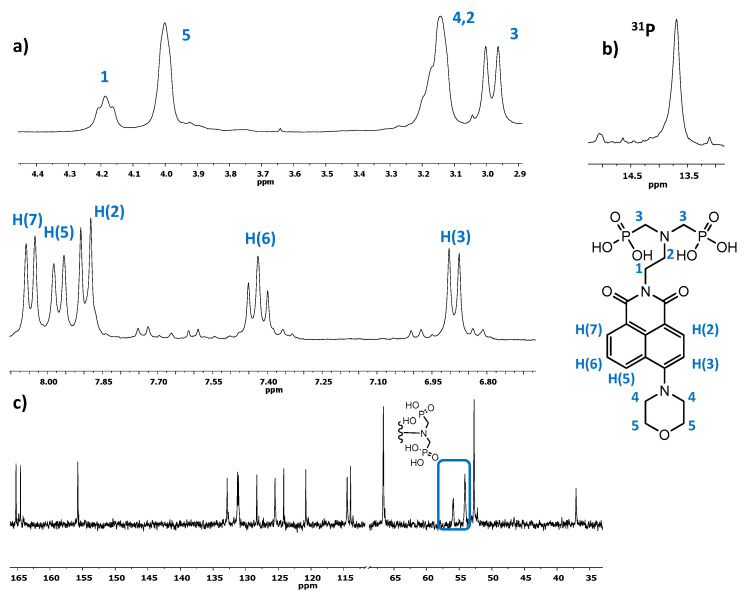
^1^H (**a**), ^31^P (**b**) and ^13^C (**c**) NMR spectra of ADMP-F in DMSO-d_6_. **^1^H NMR** (D_2_O, pH = 8.0, 300.28 MHz, t = 25.0 °C): 2.94 (d, 4H, *J* = 11.61, C**H**_2_PO_3_H), 3.04–3.20 (m, 6H, C**H_2_**N(CH_2_PO_3_H)_2_, N(C**H_2_**)_2_), 3.89–4.06 (m, 4H, O(C**H_2_**)_2_), 4.15 (t, 2H, *J* = 6.89, NC**H_2_**), 6.85 (d, 1H, *J* = 8.26, H(3)), 7.39 (dd, 1H, *J*_1_ = 7.93, *J*_2_ = 7.79, H(6)), 7.85 (d, 1H, *J* = 8.26, H(2)), 7.93 (d, 1H, H(5), *J* = 7.93), 8.01 (d, 1H, H(7), *J* = 7.79); ^13^C{^1^H} NMR (D_2_O, pH = 8.0, 75.5 MHz, t = 25.0 °C): 37.1 (N**C**H_2_), 52.7 (N(**C**H_2_)_2_), 54.1 (**C**H_2_N(CH_2_PO_3_H), 55.1 (d, *J*_1_ = 144.70, *J*_2_ = 6.05(7.15)), 66.1 (O(CH_2_)_2_), 113.9, 114.5, 120.8, 124.2, 125.5, 128.3, 131.1, 131.8, 132.9, 155.8, 164.5, 165.2; ^13^P{^1^H} NMR (D_2_O, pH = 8.0, 121.56 MHz, t = 25.0 °C): 13.69 (br.s).

Compound **2** (220 mg, 0.883 mmol) was dissolved in a mixture (10 mL) of water/ethanol (90/10 *v/v*) in the presence of 5 eq. of triethylamine and 0.1 eq. of DMAP. Next the resulting solution was added to a suspension of Compound **1** (250 mg, 0.883 mmol) in ethanol (30 mL). The resulting reaction mixture was heated at reflux for 8 h. The solvent was then removed using a rotary evaporator, and the residue dissolved in water (30 mL) and extracted with EtOAc (five times × 30 mL) to remove unreacted Compound **1**. The aqueous layer was acidified to pH 4–5 with hydrochloric acid, then diluted with isopropanol (five times) and left at +5 °C overnight. The formed precipitate was filtered off, washed with isopropanol, and dried at 70 °C. The yield of Compound **3** was 267 mg (59%). The molecular structure was confirmed by NMR spectroscopy (Figure 2). The NMR peak assignment was performed on the grounds of our previous study [15]. 

ADMP-F was further studied in comparison with two other fluorescent-tagged antiscalants of different chemical natures: HEDP-F and PAA-F1 (molecular mass c.a. 4000 Da). The synthesis of HEDP-F and PAA-F1 is described in [14] and [24], respectively. 

### 2.2. ADMP-F Fluorescence Studies

The fluorescence quantum yield Φ_F;x_ (further mentioned as Φ_fl_) measurements were performed using a Varian Cary 100 UV–vis spectrophotometer and Shimadzu RF-6000 (Shimadzu) spectrofluorometer (Table 1). All measured fluorescence spectra were corrected for the nonuniformity of detector spectral sensitivity. Quinine sulfate solution in 0.5 M H_2_SO_4_ (Φ_fl_ = 0.55) was used as a reference for the fluorescence quantum yield measurements [34]. The values of Φ_fl_ were determined by applying the following Equation (1):

(1) $$\Phi_{\mathrm{fl}}=\Phi_{F,r}\frac{\left(1-10^{-Ar}\right)D_{x}\cdot n_{x}^{2}}{(1-10^{-Ax})D_{r}\cdot n_{r}^{2}}$$

where Φ_fl_ and Φ_F,r_ denote the fluorescence quantum yield of the sample and the standards, respectively; *Ax* and *Ar* are the absorbances of the sample and the standard solutions at the excitation wavelength, respectively; *D_x_* and *D_r_* correspond to the integrated area of the emission fluorescence spectra of the sample and standards, respectively, corrected for the blank solvent; and *n_x_* and *n_r_* are the solvent refractive indices of the sample and the standards, respectively. All fluorescent-tagged antiscalants used in this study revealed fluorescence in the blue (HEDP-F, PAA-F1) or green (ADMP-F) region of the spectrum (Table 1). 

Although HEDP-F and PAA-F1 revealed fluorescence in the blue region of the spectrum (Table 1), the fluorescent channel was assigned the green pseudo-color because the dynamic range of human color perception for green is much wider than for blue. Thus, in our report, the original blue emission of HEDP-F and PAA-F1 was changed for an artificial green pseudo-color.

### 2.3. Scale Inhibition Tests

The widely accepted NACE procedure for testing scale inhibition efficacy [31] suggests that calcium sulfate and calcium carbonate solutions are supersaturated “all at once” by mixing corresponding concentrated calcium chloride and sodium sulfate or carbonate brines. While this is a good fit for oilfield antiscalant applications, it is not the case for most other water treatment technologies. However, in the rest of the common industrial processes that suffer from scale formation, supersaturation is achieved by a steady increase in the concentration of a sparingly soluble salt from an undersaturated aqueous solution to a supersaturated one either by water evaporation (cooling towers, evaporation plants) or by mechanical water removal (reverse osmosis). Therefore, it was of particular interest to study the visualization of antiscalants during a gradual supersaturation process. In order to achieve the objective of this study, a model of carbonate scum formation and gypsum fouling during the evaporation of an unsaturated solution was tested.

#### 2.3.1. Calcium Carbonate Deposition Test

Calcium carbonate deposition models the carbonate scale formation with a gradual transition from a homogeneous undersaturated solution to a supersaturated one at elevated temperature and constant aqueous phase volume. The experimental design was similar to that described in [35]. The Ca(HCO_3_)_2_ solution was obtained by the saturation of an aqueous CaO suspension with CO_2_ and subsequent removal of the solid phase by filtration (0.45 µm Millipore Nylon filter). The resultant homogeneous solution corresponded to 12.6 mmol·dm^−3^ Ca(HCO_3_)_2_, pH 7.26. Then, 100 mL of this stock solution was mixed with 0.5 mL of either antiscalant solution or distilled water (blank experiment) and then kept in a closed glass vessel under constant stirring at 70 ± 5° for 1 h, cooled, separated from CaCO_3_ deposits by filtration, and analyzed for residual calcium content. The scale inhibitor concentration was 10 mg·dm^−3^ in all runs. The heating led to some release of CO_2_. This resulted in a pH increase of up to 8.2 (blank run) and from 8.0 to 8.3 for various antiscalants. Thus, the supersaturation state for CaCO_3_ was achieved, and some deposits formed at a constant solution volume. The degree of supersaturation was estimated as SI = [Ca^init^]/[Ca^res^] = 21, where [Ca^init^] corresponded to the initial content of calcium ions in the aqueous phase of the blank solution, and [Ca^res^] indicated the calcium residual concentration in the same solution after deposits formed. The antiscalant’s efficacy ***I*** was then estimated according to the following Equation (2):

(2) $$I=\frac{C_{Ca}{}^{ing}-C_{Ca}{}^{blank}}{C_{Ca}{}^{total}-C_{Ca}{}^{blank}}\times 100$$

where $C_{Ca}{}^{total}$ is the initial concentration of calcium in the aqueous phase (mg·dm^−3^);$\;C_{Ca}{}^{ing}$ is the final concentration of calcium in the aqueous phase in the presence of antiscalant (mg·dm^−3^); and $C_{Ca}{}^{blank}$ is the final concentration of calcium in the aqueous phase in the blank run (mg·dm^−3^).

**Figure 3:**
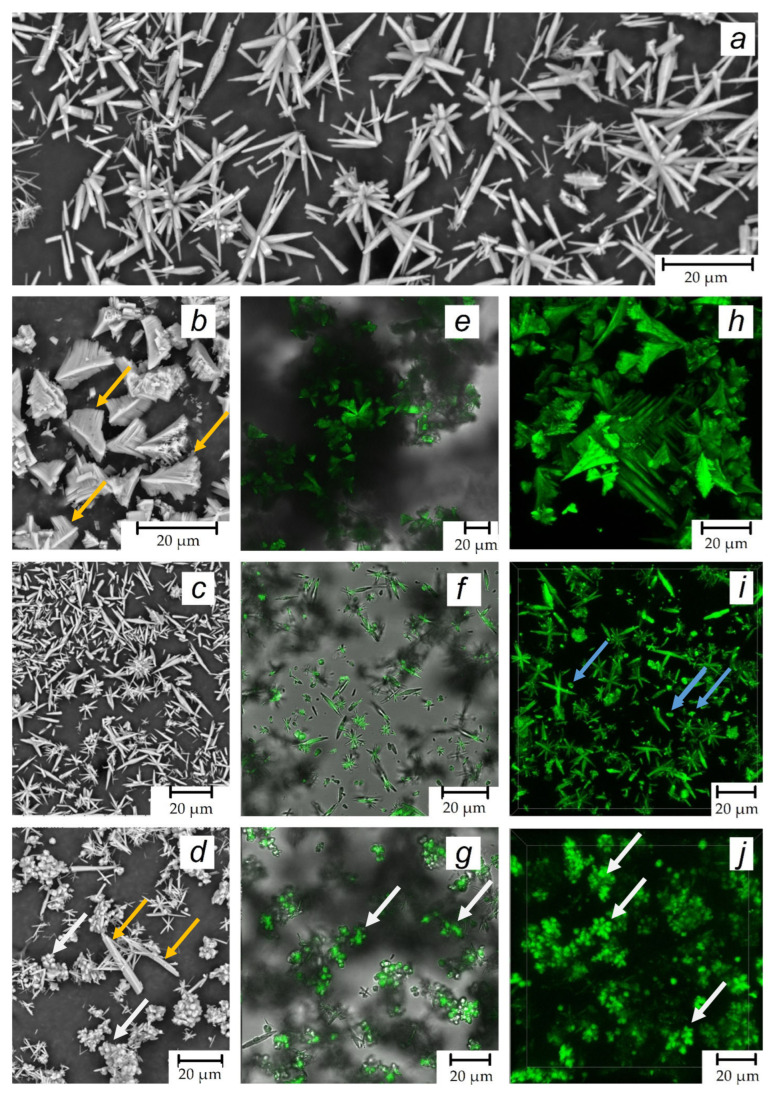
SEM (**a**–**d**), LSM 2D (**e**–**g**) and LSM 3D (**h**–**j**) images of calcium carbonate scale deposited after heating of an aqueous solution of calcium bicarbonate at 70 °C in a blank run (**a**) and in presence of 10 mg·dm^−3^ ADMP-F (**b**,**e**,**h**), HEDP-F (**c**,**f**,**i**) and PAA-F1 (**d**,**g**,**j**); (**a**–**d**,**h**–**j**) images correspond to isolated solids, and e,f,g images correspond to aqueous solutions; blue arrows indicate Ca-HEDP-F particles, white arrows indicate vaterite phase, and red arrows correspond to calcite.

The solid phase was gently rinsed with distilled water, dried at ambient temperature, and then analyzed by luminescent confocal laser scanning microscopy (LSM), scanning electron microscopy (SEM) and X-ray diffraction. The liquid phase was analyzed for Ca content (titration with EDTA) and by confocal luminescent microscopy. The luminescent and electron microscopy images are presented in Figure 3, and scale inhibition efficacy is given in Table 2, along with data on Aminat-K, which was taken as a reference compound [36,37].

Figure 3 demonstrates the perfect visualization of ADMP-F, HEDP-F and PAA-F1 on the surface of calcium carbonate. The XRD phase composition of the deposit (% *w/w*) corresponded to aragonite 68% and calcite 32% in the blank run (Figure 3a, Table 3); calcite 100% in the presence of ADMP-F (Figure 3b,e,h; Table 3); aragonite 75% and calcite 25% in the presence of HEDP-F (Figure 3c,f,i; Table 3); and calcite 50% and vaterite 50% in the presence of PAA-F1 (Figure 3d,g,j; Table 3). 

It is noteworthy that ADMP-F and PAA-F1 alter the crystalline habit of calcium carbonate, whereas HEDP-F does not appear to have any effect on it (Table 3). The same behavior was detected earlier for non-fluorescent bisphosphonate HEDP in a NACE test [36] (Table 3). Although the degree of supersaturation, the way in which supersaturation was achieved, and the final pH were different in the recent study and in the NACE tests, there is some qualitative agreement between commercial antiscalants and their fluorescent analogs. Indeed, both PAA and PAA-F1 partially stabilized vaterite, while ATMP and ADMP-F stabilized calcite, and HEDP and HEDP-F seemed to be indifferent in this respect. 

The ability to modify the crystal lattice of calcium carbonate qualitatively correlates with scale-inhibiting efficacy (Table 2): Aminat-K > PAA-F1 > ADMP-F >> HEDP-F. As a matter of fact, the least efficient antiscalant HEDP-F does not change the crystal habit of calcium carbonate, whereas the more efficient antiscalants, Aminat-K, ADMP-F and PAA-F1, induce such changes.

Another important observation relates to the location of the antiscalant on the calcium carbonate surface. ADMP-F covers the entire surface of the deposit, represented by calcite (100%) (Figure 3e,h). HEDP-F also covers the surface of calcite and aragonite crystals but leaves some surfaces “free” of scale particles (Figure 3f), while PAA-F1 is localized exclusively on the vaterite phase (spherical particles), and elongated particles of calcite reveal no luminescence (Figure 3g,j). Therefore, PAA-F1 seems to have a particularly high affinity for the vaterite surface, and its sorption stabilizes this metastable polymorph, which can easily be transformed into the stable calcite phase [38]. A similar result of the least stable form of calcium carbonate stabilization was found for polyaspartate (PASP) [36,39], polyepoxysuccinate (PESA) [39], some other polymers [40], and EDTA [38]. Vaterite is the first phase known to form from supersaturated CaCO_3_ solutions [41]. Then, in the absence of PAA-F1, vaterite is completely transformed into the more stable calcite and aragonite phases (Table 3), while the presence of this antiscalant inhibits vaterite decomposition, it accelerates the transition of aragonite to calcite. Notably, ADMP-F does not retard vaterite–calcite and aragonite–calcite transformations. 

On the other hand, there is no evidence that antiscalants are specifically located at kink, step, or selected edge sites of growing carbonate crystals, as is expected within the framework of conventional scale inhibition mechanisms [4]. A highly effective antiscalant PAA-F1 is distributed on the vaterite surface, uniformly covering the entire crystal surface, as is ADMP-F (Figure 3e,h). However, unlike ADMP-F, PAA-F1 leaves almost half of the total mass of CaCO_3_ crystals (those that form calcite) uncovered. Meanwhile, the least effective antiscalant HEDP-F uniformly covers the entire crystal surface of isolated calcite and aragonite phases much better than PAA-F1 (Figure 3i). Thus, there is no direct correlation between the effectiveness of antiscalant and its ability to be adsorbed on the crystal surface.

**Figure 4:**
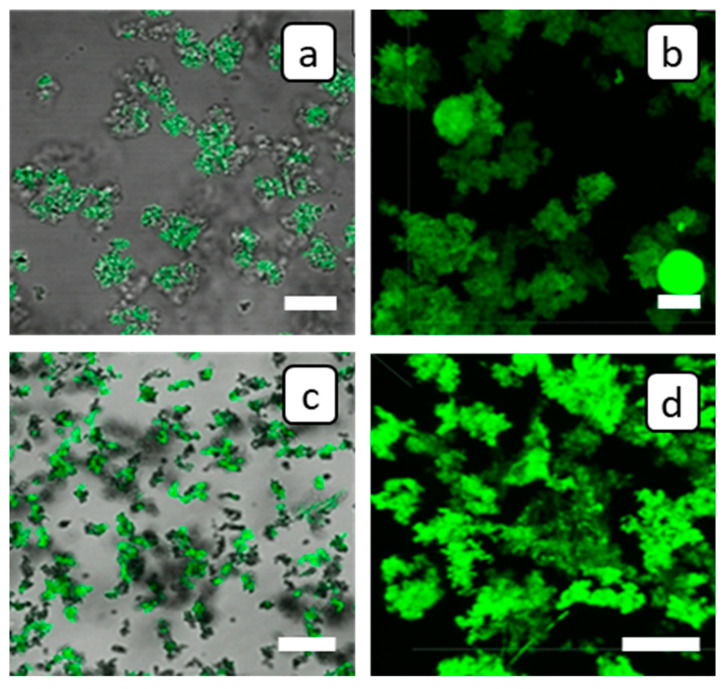
LSM 2D (**a**,**c**) and 3D (**b**,**d**) images of solids deposited after 20-fold evaporation of 20 mg·dm^−3^ CaCl_2_ aqueous solution at 70 °C in presence of 10 mg·dm^−3^ HEDP-F (**a**,**b**) and ADMP-F (**c**,**d**). Scale bar 20 µm.

At the same time, some of the HEDP-F molecules are concentrated in a separate phase (Ca-HEDP-F) in the form of tiny spherical particles (Figure 3i, indicated by red arrows). Notably, ADMP-F is also prone to the formation of insoluble calcium salts (Ca-ADMP-F). In order to make sure that this was the case, a blank supplement experiment was performed. To achieve the objective, 500 mL of 20 mg·dm^−3^ CaCl_2_ aqueous solution was evaporated in a rotary evaporator to 25 mL residual volume at 70 °C in the presence of antiscalants (10 mg·dm^−3^). Indeed, under these conditions, both Ca-ADMP-F (Figure 4a,b) and Ca-HEDP-F (Figure 4c,d) stand out in the form of crystals, whereas PAA-F1 does not form a solid phase.

Notably, the scale inhibition activity under the conditions selected for this study appears to be much lower than that estimated by the NACE test. Indeed, PAA-F1 shows only a 26% inhibition compared to 49% registered by the NACE test [24] for the same antiscalant dosage. The same is also valid for Aminat-K (ATMP), which, according to NACE [36], showed an 80–100% efficacy at doses of 1–3 mg·dm^−3^ and only a 40% inhibition at 10 mg·dm^−3^ in the present work (Table 2). Apart from the issues of different ways of reaching supersaturation, such a difference could also be explained by the sufficiently harsher conditions in our experiments compared to the NACE test: a higher supersaturation in our experiments (21 versus 1.5) and a higher pH (>8 versus <7 according to the NASE test). 

#### 2.3.2. Calcium Sulfate Deposition Test

Gypsum deposition experiments imitated evaporation plant conditions with a gradual transition from an undersaturated to a supersaturated solution at elevated temperatures. A small molar excess of chalk was mixed with sulfuric acid, the mixture was allowed to equilibrate at room temperature for 24 h, and the solid phase was then removed by filtration (0.45 µm Millipore Nylon filter). The aqueous phase was a 0.0167 mol·dm^−3^ saturated solution of gypsum (669 mg·dm^−3^ calcium) at pH 7.2, which corresponds well to the solubility limit of CaSO_4_·2H_2_O [42]. This solution was used for further experiments. Next, a 100 mL sample of this stock solution was mixed with 0.5 mL of either antiscalant aqueous solution or with 0.5 mL of distilled water (blank experiment). This operation made the saturated gypsum solution an undersaturated one. This mixture was then evaporated in a water bath at 70 ± 5 °C until the total volume of the solution was reduced to 50 mL, either in the presence of 10 mg·dm^−3^ of antiscalant or without a scale inhibitor (blank experiment), cooled up to 25 °C, equilibrated for 24 h, filtered, and then the aqueous phase was analyzed for residual calcium content. Thus, the supersaturation (SI) in all runs was 2. Here the supersaturation is denoted as SI = [Ca^total^]/2, where [Ca^total^] corresponds to the initial concentration of calcium ions in the aqueous phase, which is nearly the same as the calcium concentration according to the gypsum solubility limit at 25 °C. The antiscalant’s efficacy ***I*** was then estimated according to the following Equation (3):

(3) $$I=\frac{0.5\;C_{Ca}{}^{ing}-0.5\;C_{Ca}{}^{blank}}{C_{Ca}{}^{total}-0.5\;C_{Ca}{}^{blank}}\times 100$$

where $C_{Ca}{}^{total}$ is the initial concentration of calcium in the aqueous phase (mg·dm^−3^);$\;C_{Ca}{}^{ing}$ is the final concentration of calcium in the aqueous phase in the presence of antiscalant (mg·dm^−3^); and $C_{Ca}{}^{blank}$ is the final concentration of calcium in the aqueous phase in the blank run (mg·dm^−3^).

The solid phase was gently rinsed with distilled water, dried at ambient temperature, and then analyzed by LSM, SEM and X-ray diffraction. The liquid phase was analyzed for Ca content (titration with EDTA) and by LSM.

Experiments with the evaporation of calcium sulfate solution resulted in a similar scale inhibiting efficacy sequence relative to calcium carbonate: Aminat-K ~ PAA-F1 > ADMP-F > HEDP-F. At the same time, the sequence PAA-F1 > HEDP-F is also in good agreement with the NACE tests [15,24] (Table 2), although the HEDP-F location appears to be very different in the NACE test [15] and the present work. Indeed, in the former case, HEDP-F was observed mainly inside and partly on the surface of CaSO_4_·2H_2_O crystals [15], while in the present work, it was not detected either inside or on the surface of gypsum, as in the experiment at ambient temperature [15] (see the explanations below). Meanwhile, the absolute values of HEDP-F and PAA-F1 efficacy are rather close to those indicated in the NACE test, with almost the same degree of supersaturation as in our experiments (2 versus 1.43).

**Figure 5:**
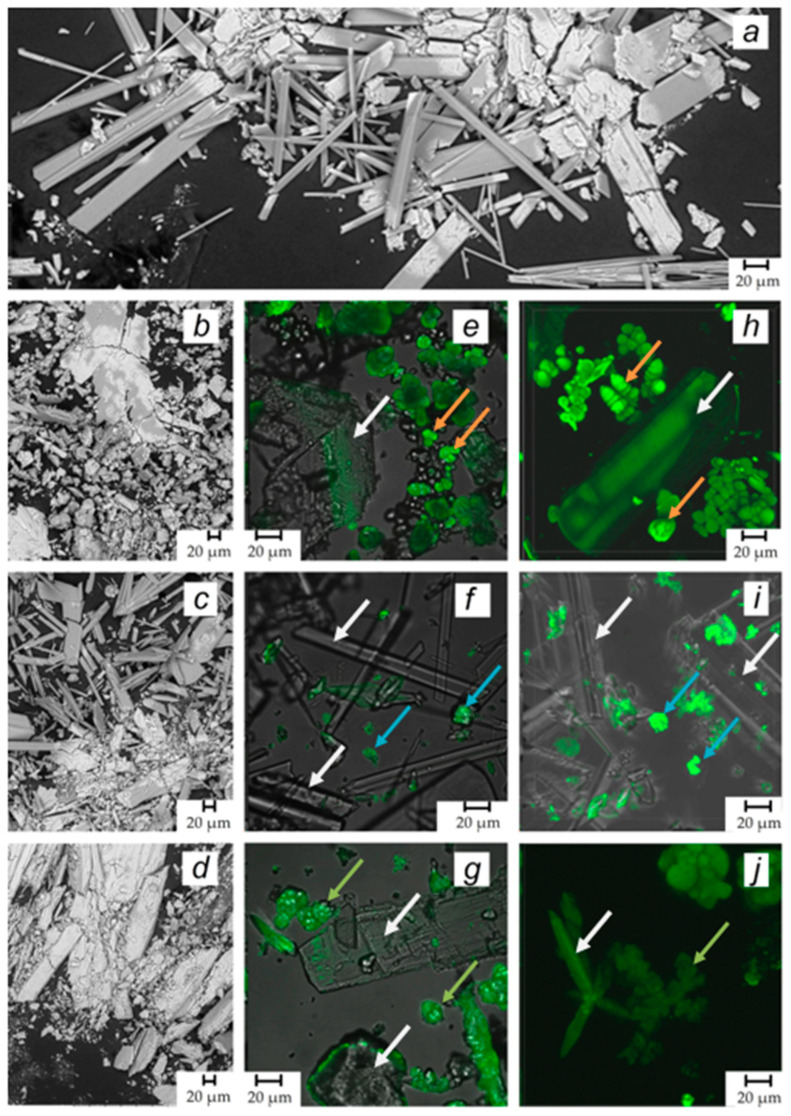
SEM (**a**–**d**), LSM 2D (**e**–**g**) and LSM 3D (**h**–**j**) images of gypsum, deposited after evaporation of an undersaturated aqueous solution at 70 °C in a blank run (**a**) and in presence of 10 mg·dm^−3^ ADMP-F (**b**,**e**,**h**), HEDP-F (**c**,**f**,**i**) and PAA-F1 (**d**,**g**,**j**); (**a**–**d**,**h**–**j**) images correspond to isolated solids, and (**e**–**g**)—to aqueous solutions; red arrows indicate Ca-ADMP-F particles, blue arrows show Ca-HEDP-F species, green arrows indicate presumably Ca-PAA-F-1 phase, while white arrows—gypsum phase.

According to the XRD analysis, exclusively the gypsum (CaSO_4_·2H_2_O) crystals deposition was detected both in the blank run and in the presence of antiscalants. However, in the presence of ADMP-F and PAA-F1, a noticeable change in crystal shape was observed: gypsum crystals became less elongated (Figure 5b,d). At the same time, HEDP-F demonstrated no change in the habit of the CaSO_4_·2H_2_O crystals. Moreover, it was not detected at all on the surface of gypsum crystals (Figure 5f). A similar effect was previously observed for HEDP-F [15] in the case of “all at once” artificially arranged supersaturation by mixing concentrated calcium chloride and sodium sulfate brines at ambient temperature. In both cases, HEDP-F is likely to form its own separate phase with calcium ions: Ca-HEDP-F. It can be seen as small green spherical spots in Figure 5f,i. In order to confirm this assumption, an auxiliary experiment was carried out with CaCl_2_ evaporation in the presence of HEDP-F under the same conditions as for gypsum evaporation (Figure 4a,b). Indeed, fluorescent solids with a similar shape as in Figure 5i and in [15] have been detected. 

**Figure 6:**
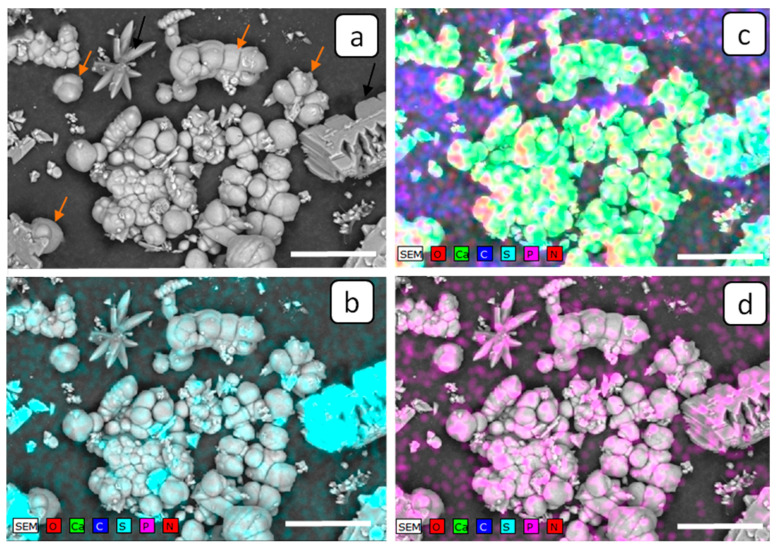
SEM image (**a**) and SEM-EDS map of element distribution for calcium sulfate isolated in presence of ADMP-F: distribution of sulfur (**b**), of all elements (**c**) and of phosphorus (**d**); gypsum particles are indicated by black arrows and Ca-ADMP-F phases by red arrows; scale bar 300 µm.

A paradox phenomenon, first reported for HEDP-F in [15], is again reproduced, but for evaporation plant conditions: the antiscalant does not interact with the scale but still provides inhibition. ADMP-F is also found to form its own insoluble phase Ca-ADMF-F, which is present in Figure 5e,h. For a better assignment of spherical particles to the Ca-ADMF-F phase, SEM-EDS mapping was performed (Figure 6). Indeed, all spherical species revealed the presence of P, Ca and N, but no sulfur was detected. Alternatively, the macrocrystals of gypsum indicate Ca, S and traces of antiscalant (P and N elements). Thus, unlike HEDP-F, ADMP-F covers the surface of some gypsum crystals (Figure 6d), while others remain free of antiscalant molecules (Figure 5e,h). PAA-F1 also leaves some CaSO_4_·2H_2_O crystals uncovered, while others are definitely covered by the antiscalant. In summary, incomplete coverage of the gypsum scale by ADMP-F (Figure 5e) and 75% inhibition contrasts with the complete coverage of the calcium carbonate deposit surface by its molecules and only 15% inhibition (Figure 3e). On the other hand, complete coverage of the calcium carbonate surface by ADMP-F does not make it a more effective antiscalant than PAA-F1, which only covers the vaterite particles (50% of the total deposit). These facts provide evidence in favor of the hypothesis that the sorption of antiscalant on the sparingly soluble salt crystal surface plays a secondary role in the overall inhibitory effect.

In the case of PAA-F1, the solid phase was sparse (99% inhibition), and it was rather difficult to identify individual crystals and their shape (Figure 5d,g,j). In the liquid phase, the semispherical solids and prismatic crystals could be distinguished. The former show luminescence and may belong either to the highly distorted gypsum phase, covered by antiscalant, or to the Ca-PAA-F1 species, similar to those found in [21].

## 3. Discussion

The precipitation of a crystalline substance from a bulk solution to the site of scale formation requires supersaturation (SS). SS occurs when a solution is concentrated beyond the solubility limits of one or more of its constituents [4,8]. Supersaturation may result from several conditions: (i) heating/cooling (cooling a normally soluble salt solution or heating an inversely soluble salt solution may result in supersaturation); (ii) water evaporation (in water cooling towers, evaporation plants); (iii) separation of pure water at ambient temperature (in membrane processes); (iv) solution mixing, when a soluble salt is added to a solution of another salt, which has a common ion or produces a sparingly soluble salt, the solubility limit can be exceeded because the ionic product becomes greater than the solubility constant (oilfield applications); (v) alteration of the carbonate/bicarbonate equilibrium, either by changing the pH or affecting the equilibrium conditions of the carbon dioxide dissolved from the atmospheric air (in the cooling tower). Once SS is achieved, multistep scale formation pathways take place, including steps such as nucleation, growth, aggregation, self-assembly, sedimentation, and sometimes structural rearrangement of the sediment [2,4,43,44,45,46]. Antiscalants can affect all of these steps in one way or another, and antiscalant visualization provides a unique opportunity to differentiate the effect of scale inhibitors on the corresponding step in specific cases. 

Three antiscalants of different natures were used in our experiments: aminophosphonate (ADMP-F), bisphosphonate (HEDP-F) and polyacrylate (PAA-F1). They were tested in calcium carbonate and gypsum deposition processes initiated by continuous saturation of initially unsaturated aqueous solutions of these salts at elevated temperatures. The experimental conditions are, therefore, different from those proposed for the NACE test (supersaturation is achieved immediately by mixing calcium and carbonate or sulfate salts). Nevertheless, there is a qualitative correlation in the order of reagent effectiveness between continuous saturation (present work) and supersaturation provided “all at once” (NACE test) for both calcium carbonate and gypsum deposits: PAA-F1 > ADMP-F > HEDP-F. Notably, there was a lack of correlation between the NACE results and the estimates previously obtained on the basis of induction time measurements [47].

In all cases, an antiscalant inhibited scale formation and was detected on the surface of the deposit, with the sole exception of the HEDP-F/gypsum system. Furthermore, the more efficient reagents, ADMP-F and PAA-F1, affect the calcium carbonate crystal modification and the morphology of the gypsum crystals, whereas the least efficient, HEDP-F, did not change either the crystal form of CaCO_3_ or the crystal habit of CaSO_4_·2H_2_O. In this relevance, there is also a correlation between inhibition efficacy and the ability to change the crystal lattice in a particular case. In general, however, this is not the case, as numerous examples of the opposite trend have been described [9]. At the same time, in this study, there was no correlation between the coverage of the crystal surface by the antiscalant and the efficacy of the antiscalant. In fact, the least effective inhibitor for CaCO_3_ (HEDP-F) covered the entire surface of the calcium carbonate deposit, while the most effective (PAA-F1) left half of the solid phase surface (calcite fraction) free. Apparently, the antiscalant covers the scale surface after macrocrystal formation. This phenomenon is likely to be responsible for secondary crystal form transformation, e.g., stabilization of vaterite by PAA-F1 but is unlikely to affect the rate of crystal deposition. Furthermore, the visualization of the antiscalant molecules did not provide any evidence that the antiscalants are specifically located at the kink, step, or selected ledge sites of growing carbonate crystals, as expected within the framework of conventional scale inhibition mechanisms [4]. All these facts support the hypothesis that the sorption of the antiscalant on the crystal surface of the sparingly soluble salt plays a secondary role, if any, in the total inhibitory effect. This is further supported by the detection of the Ca-ADMP-F and Ca-HEDP-F phases together with gypsum macrocrystals. Thus, it appears that sufficient amounts of these phosphonates are partially (ADMP-F) or completely (HEDP-F) excluded from interaction with gypsum but still provide scale inhibition. 

The most interesting result was registered for the HEDP-F/gypsum system. HEDP-F retarded the deposition of gypsum scale but did not interact with it, as this antiscalant was not detected at all on the surface of the gypsum crystals. A similar effect has recently been reported for HEDP-F in two cases at ambient temperature: in the case of “all at once” artificial supersaturation by mixing concentrated calcium chloride and sodium sulfate brines [15] and in a reverse osmosis experiment with a steadily increasing gypsum concentration [16]. This is the first time such an effect has been observed under evaporation conditions. In all three cases, HEDP-F formed its own separate phase with calcium ions (Ca-HEDP-F) in the form of tiny spherical particles, while the crystal form or habit of calcium sulfate was not affected, and no traces of fluorophore were detected either inside or on the surface of the gypsum. This contradicts the generally accepted notion of “antiscalant—sparingly soluble salt” interactions [2,4,6], which relates the scale inhibition phenomenon precisely to antiscalant sorption on the surface of sparingly soluble salt crystals. 

However, all the aforementioned experimental facts have a rather clear explanation if the nucleation step is considered as the rate-determining step, and the dominant mechanism of antiscalant activity is shifted from the “antiscalant—sparingly soluble salt” interactions plane to the “antiscalant—particulate matter” plane. Nucleation is defined as the process by which the smallest stable aggregates of a crystalline phase are formed in a crystallizing system. In bulk aqueous solutions, the naturally occurring solid impurities (heteronuclei particulates) are recognized to act as primary crystallization centers [2,43,44]. On the grounds of our previous studies of gypsum scaling [9,15,20,21], we suppose that the major contribution to scale inhibition is associated with the nucleation step. It was demonstrated that bulk nucleation in supersaturated solutions of sparingly soluble salts takes place on natural solid nanoimpurities, inevitably present in any aqueous solution [9,15,16,21]. An antiscalant blocks the surface of these nanoimpurities and thus hinders the nucleation step by reducing the number of potential nucleation centers. In our recent study, the monotonic transition from an undersaturated to a supersaturated state of CaCO_3_ and CaSO_4_ solutions provides an excellent opportunity for such a blockage. Scale inhibitor sorption occurs in competition with Ca^2+^ and either SO_4_^2−^ or CO_3_^2−^ ions. Those particles that are able to preferentially adsorb these ions lead to further carbonate or sulfate scale formation, while those covered by the antiscalant are excluded from scale formation. In the case of HEDP-F and ADMP-F, this process results in the formation of the Ca-HEDP-F and Ca-ADMP-F phases. 

At the same time, an excess of antiscalant may also provide some coverage of the surfaces of the calcium carbonate and calcium sulfate nuclei, reducing their growth rate in different ways for the different crystal faces. This leads to changes in crystal morphology. However, we consider this contribution to be of secondary importance to the overall scale inhibition effect. Meanwhile, such secondary sorption of PAA-F1 on calcium carbonate nuclei provides stabilization of vaterite, but this reagent is not adsorbed by the aragonite surface. Thus, antiscalant visualization provides a unique opportunity to track scale inhibitors in multiphase solids. 

Generally, ADMP-F (15% inhibition) is less effective against CaCO_3_ scale formation relative to the same mass of industrial Aminat-K/ATMP (40% inhibition) (Table 2). At the same time, this difference between Aminat-K/ATMP (99% inhibition) and ADMP-F (73% inhibition) is much less pronounced for gypsum scale formation under evaporation conditions. However, the aforementioned differences are not as great when the same molecular masses (MM) of ATMP (MM 299) and ADMP-F (MM 509) are considered. Obviously, the lower amount of phosphonate groups in the ADMP-F molecule relative to ATMP leads to lower antiscaling efficacy. On the other hand, ADMP-F is not elaborated as an alternative to ATMP and other aminophosphonates but as a tracer of these reagents, which is capable of enabling their monitoring in industrial water treatment in “online” mode, and as a tool of aminophosphonate visualization in scale formation mechanism studies. 

In summary, the key conclusions of this study are:

(i). The novel fluorescent antiscalant ADMP-F demonstrates excellent visualization capability and good inhibition activity against both CaCO_3_ and CaSO_4_·2H_2_O scales formed under conditions of slow supersaturation. Thus, ADMP-F may become a promising tracer of non-fluorescent organophosphonate scale inhibitors, such as ATMP or HEMPA, in various water treatment applications and a powerful tool for studying the mechanisms of scale inhibition phenomena.
(ii). The visualization of three fluorescent-tagged antiscalants of different natures during calcium carbonate and gypsum scale formation provided a unique opportunity to gain insight into inhibition mechanisms. Contrary to popular belief, there is no evidence to suggest that antiscalants are specifically located at kink, step, or on selected edge sites of growing calcium carbonate and gypsum crystals. Furthermore, there was no direct correlation detected between the effectiveness of the antiscalant and its ability to be adsorbed on the crystal surface, as is expected within the framework of conventional scale inhibition mechanisms. A paradoxical effect, which was first reported for HEDP-F in 2019, for gypsum crystallization at ambient temperature was reproduced again, but for evaporation plant conditions: the antiscalant does not interact with the scale but still provides inhibition.
(iii). Phosphorus-based antiscalants have been found to form their own phases with calcium ions. Most of these inhibitors have been shown to accumulate in such species but not on the surface of scale crystals. Despite this, scale inhibition will still take place, but the degree of success depends on how the antiscalant is injected. If the scale inhibitor is injected primarily into the sodium sulfate or sodium carbonate solution, it is expected to be more effective than if it is injected primarily into the calcium chloride solution. Therefore, this effect has to be taken into account in antiscalant screening procedures. In any case, it should become a standard practice to specify the method of antiscalant injection in any publication on the evaluation of the efficacy of scale inhibitors.
(iv). This present study demonstrates that antiscalants of different natures reveal different behaviors and different locations on the deposit surfaces. Nevertheless, a universal explanation encompassing all these cases is possible if bulk heteronucleation is assumed. It takes place on natural solid nanoimpurities, which are inevitably present in any aqueous solution. An antiscalant blocks the surface of these nanoimpurities, thus hindering the nucleation step by reducing the number of potential nucleation centers.
(v). A comparison of the efficacy of antiscalants, estimated within the framework of different static tests, based on different ways of achieving supersaturation has shown that for particular cases of calcium carbonate scum formation and gypsum deposition within the evaporation process, the same ranking of antiscalants is obtained as in the internationally recognized static NACE test, although the mechanisms of scale formations in the latter case are different. However, the validity of dynamic tests for the same processes seems unclear and requires further study.

## 4. Materials and Methods

The commercial antiscalant Aminat-K was purchased from the company TRAVERS, Moscow, Russia. This reagent contains ATMP as an active substance. It was used as received, and its dosages were calculated with reference to the ATMP content. Chemicals of reagent quality were used for the preparation of calcium carbonate and calcium sulfate solutions. After rinsing with deionized water and air drying at 50 °C, the precipitated solids were characterized by SEM (Hitachi TM-3030) and powder XRD (Bruker D8 Advance diffractometer; Cu KD; Ni-filter; LYNXEYE detector). The XRD phase identification was completed using the Joint Committee on Powder Diffraction Standards (JCPDS) database, and the relative phase content was estimated with Topaz R software (Bruker AXS). The sample examinations by SEM were performed at a 15 kV accelerating voltage in a charge-up reduction mode with the crystal phase located on a conducting double-sided tape. Energy-dispersive X-ray spectroscopy (EDS) data was processed using QUANTAX 70 software. The system was calibrated using a copper standard. EDS data was acquired in Spot Mode and Mapping Mode. Element coloring was used in Automatic Mode. Spot Mode was used for quantitative element analysis and Mapping Mode for element distribution localization.

Fluorescent microscopy measurements were performed with a laser scanning confocal microscope LSM-710-NLO (Carl Zeiss Microscopy, Jena, Germany) at a ×20 Plan-Apochromat objective (NA = 0.8) for both liquid and solid samples. The fluorescence of antiscalants was recorded in the wavelength range of 470–600 nm when excited by a laser with a wavelength of 458 nm. The 3D fluorescence images were recorded with a step 1 µm along the *Z* axis. The liquid samples were placed onto a Petri dish with a 0.16 mm thick glass bottom and were presented as 2D images, while the solids were presented as 3D images. The 3D fluorescence images were recorded with a step 1 µm along the *Z* axis.

## Figures and Tables

**Table 1:** Absorption and emission of fluorescent-tagged antiscalants ^1^.

Antiscalant	*λ*_max_, nm	*λ*_max_^fl^, nm	Φ_fl_, %	Ref.
ADMP-F	401	545	13	Present work
HEDP-F	375	460	86	[15]
PAA-F1	375	465	69	[24]

^1^ Measurements were performed in H_2_O for 1.5 × 10^−5^ mol·dm^−3^ of ADMP-F and HEDP-F.

**Table 2:** Scale inhibition in slow saturation runs at 10 mg·dm^−3^ antiscalant dosage.

Compound	CaCO_3_ Scaling		CaSO_4_ Scaling			
	Inhibition, %	pH ^1^	Present Work		NACE	
			Inhibition, %	pH ^1^	Inhibition, %	pH ^1^
Blank	0	8.2	0	8.0	0	6 to 7
ADMP-F	15 ± 1	8.3	73 ± 2	8.2	-	6 to 7
HEDP-F	1.0 ± 0.5	8.5	16 ± 1	8.1	78 ^2^ [15]	6 to 7
PAA-F1	26 ± 2	8.0	99 ± 2	8.1	73 ^3^ [24]; 92 ^4^ [24]	6 to 7
Aminat-K	40 ± 5	8.4	99 ± 1	8.4	100 ^5^ [37]	6 to 7

^1^ pH, measured at the end of the experiment; ^2^ 25 mg·dm^−3^ dosage; ^3^ 10 mg·dm^−3^ dosage; ^4^ 15 mg·dm^−3^ dosage; ^5^ 3 mg·dm^−3^ dosage of ATMP.

**Table 3:** XRD calcium carbonate scale characterization results.

Sample	Antiscalant Concentration, mg·dm^−3^	Solid Phase Composition ^1^, % *w/w*			Ref.
		Calcite	Aragonite	Vaterite	
Blank	0	32	68	-	Present work
	0	82	18	-	NACE test, [36]
PAA	10	40	60	-	NACE test, [36]
	5	13	77	10	
PAA-F1	10	50	-	50	Present work
HEDP	5	82	18	-	NACE test, [36]
HEDP-F	10	25	75	-	Present work
ATMP	5	18	82	-	NACE test, [36]
	1	100	-	-	
ADMP-F	10	100	-	-	Present work

^1^ The accuracy of a relative particular phase content is within 10–15%.

## Data Availability

Not applicable.

## Abbreviations

ATMP	Aminotris(methylenephosphonic acid)
ADMP-F	2-(6-Morpholino-1,3-dioxo-1H-benzo[de]isoquinolin 2(3H)yl)ethylazanediyl)bis(methylenephosphonic acid)
Ca-ADMP-F	Calcium salt of ADMP-F
PAA	Polyacrylate
PAA-F1	Polyacrylate with an implemented 6-methoxy-1,3-dioxo-1H-benzo[de]isoquinolin-2(3H)-yl fluorescent fragment
HEDP	1-Hydoxyethylydene-1,1-bisphosphonic acid,
HEDP-F	1-Hydroxy-7-(6-methoxy-1,3-dioxo-1H-benzo[de]isoquinolin-2(3H)-yl)heptane-1,1-diyldi(phosphonic acid)
Ca-HEDP-F	Calcium salt of HEDP-F
HEMPA	Hydroxyethylamino-di(methylene phosphonic acid)
Aminat-K	Industrial antiscalant with ATMP as an active substance
SEM	Scanning electron microscopy
EDS	Energy-dispersive X-ray spectroscopy
